# The importance of socioeconomic position in smoking, cessation and environmental tobacco smoke exposure during pregnancy

**DOI:** 10.1038/s41598-020-72298-8

**Published:** 2020-09-24

**Authors:** Joana Madureira, Alexandra Camelo, Ana Inês Silva, Ana Teresa Reis, Filipa Esteves, Ana Isabel Ribeiro, João Paulo Teixeira, Carla Costa

**Affiliations:** 1 Environmental Health Department, National Institute of Health, Rua Alexandre Herculano 321, 4000-055 Porto, Portugal; 2 EPIUnit - Instituto de Saúde Pública, Universidade do Porto, Rua das Taipas 135, 4050-600 Porto, Portugal; 3 ICBAS-Institute of Biomedical Sciences Abel Salazar, University of Porto, Rua de Jorge Viterbo Ferreira 228, 4050-313 Porto, Portugal

**Keywords:** Risk factors, Public health

## Abstract

Tobacco is still a leading cause of premature death and morbidity. Particular attention has been given to pregnant women due to the scientific evidence on the importance of early life exposures for disease onset later in life. The purpose of this study was to assess smoking prevalence, smoking cessation rate and environmental tobacco smoke (ETS) exposure, and the role of socioeconomic position (SEP) on these behaviors among pregnant women. Cross-sectional data of 619 pregnant women, aged between 18 and 46 years, from Porto Metropolitan Area, Portugal, on current smoking, ETS exposure and SEP indicators was collected, face-to-face, using a questionnaire filled in during a personal interview at the postpartum hospital stay. The smoking prevalence, and ETS exposure among non-smokers before pregnancy was 27.6% and 57.4%, respectively. 4.1% of the participants reported to have stopped smoking before pregnancy, whereas about 41% quitted along pregnancy, resulting in a smoking prevalence at birth of 14.6%. Exposure to ETS also decreased throughout pregnancy to 49.8% at birth. Lower educational level was significantly associated with both higher smoking prevalence and exposure to ETS and lower smoking cessation. This study demonstrates that smoking and ETS exposure during pregnancy remains high, and that there are still significant socioeconomic inequalities in smoking; thus tobacco-focused preventive interventions need to be reinforced.

## Introduction

Worldwide, smoking is the leading preventable cause of human morbidity and mortality^1^. Consumption of tobacco products account for nearly 8 million deaths per year around the world^1^, and over 10,000 deaths in Portugal^2^.

According to the World Health Organization (WHO) statistics, the prevalence of smoking is still a major public health concern among women^3^. In Portugal, daily smoking remains higher in men than in women, even though prevalence has been decreasing in men (28.8% in 2005 and 26.7% in 2014) and increasing in women (11.3% and 14.6% in 2005 and 2014, respectively)^4^, these trends are expected to maintain in the next years, in some countries, diminishing the differences observed between sexes^1^.

Soon after the recognition of the health effects related to smoking, it has become evident that exposure to environmental tobacco smoke (ETS) also constitutes a health risk factor^5^ and that there is no secure threshold of exposure^6^. For this reason, ETS was classified as carcinogenic for humans (group 1) in 2012 by the International Agency for Research on Cancer^7^. Available data on ETS exposure shows that, in 2015, 12.8% of the Portuguese population was exposed to ETS either at home, in transports, public areas or in the workplace more than one hour per day^8^. A substantially higher prevalence (29.0%) was reported among non-smoking individuals considering only exposure in workplaces and leisure areas in a multi-country European study^9^.

Portugal was one of the countries that signed the WHO Framework Convention on Tobacco Control, leading to the implementation of the anti-smoking law in all enclosed public spaces, the 37/2007 law^10^, in 2008. This law contained a new framework to protect individuals from ETS, and to encourage cutting down/quitting smoking and establish further regulation for the information provided on tobacco products, packaging, and labeling, as well as further restrictions on the advertising. Despite this and the extensive information on the harmful health effects, some women continue to smoke during pregnancy^11–13^. It has been estimated that 4.2–18.9% of women in 15 European countries continue smoking during pregnancy^13^. De Wolff et al.^11^ reported a prevalence of maternal smoking in Denmark ranging from 12–22% before pregnancy to 5–10% during the pregnancy. These results are in line with prior studies carried out in Finland^12^, Norway^14^, and Iceland^15^. In Portugal, estimates from 2005–2006 indicated that 23% of Portuguese women smoked early in pregnancy while 12.0% continued to smoke during pregnancy^16^; more recent data (2017–2018) pointed to a prevalence of 14% of smoking women during pregnancy^17^. No data is yet available for ETS exposure among pregnant women in Portugal.

There is abundant evidence that patterns of tobacco exposure (use and/or ETS) are related to indicators of socioeconomic position (SEP), not only in the general population^18–21^ but also among pregnant women^11,13,22–26^. These indicators include education^11,13,18,21,23,24^; income^19–21^ and employment^11,13,26^. Moreover, the level of deprivation in the immediate neighborhood of residence seems to play a role in shaping smoking behavior^27^, underlining the existence of residential contextual effects on health related-behavioral practices.

However, given the heterogeneity in smoking prevalence across countries and regions, findings from international studies might not reflect the same patterns found in other countries, particularly in Portugal. In addition, the lack of uniform study design, data collection and study population makes it difficult to assess whether these SEP indicators for smoking and ETS exposure in pregnancy are similar in frequency and effects across countries. To fill this gap and to contribute to monitoring of smoking and ETS exposure trends and other dimensions of smoking, this study assesses smoking prevalence, smoking cessation rate and ETS exposure, as well as the role of the SEP on these behaviors among pregnant women.

## Material and methods

### Study design

Data were collected in the framework of the NeoGene project (funded by FCT/FAPESP; reference FAPESP/19914/2014), a cross-sectional birth study that aims to investigate the genetic and epigenetic effects of prenatal tobacco use and ETS exposure, considering simultaneous exposure to numerous and possibly interacting chemicals. In that respect, a wide spectrum of metals, particulate matter, polycyclic aromatic hydrocarbons and other organic compounds assume particular interest.

### Participants

The study population included pregnant women receiving prenatal care in Centro Hospitalar de São João (CHSJ). Inclusion criteria comprised women of all ages that spoke Portuguese and singleton pregnancy. Women with infectious diseases, mental and physical disabilities were excluded as well as pregnancies with fetal congenital malformations and genetic disorders. Pregnant women were invited to participate in the project between April 2017 to July 2018 during the last prenatal appointment (after week 36 of pregnancy) at CHSJ. A total of 838 pregnant women were recruited.

### Data collection and variable description

Data were collected within 72 h after delivery, during hospital stay, through individual face-to-face interviews using a paper-based structured questionnaire that was based on the previously used in different Portuguese epidemiological survey^16^. Out of the recruited women, 622 answered the questionnaire; from these, 3 participants were excluded given the missing information in smoking habits and ETS exposure, leaving data from 619 mothers for further analyses.

#### Demographic variables

Maternal characteristics were collected, including age, parity, area of residence and alcohol drinking habits. Age was categorized as ≤ 25 years old (young adulthood) and > 25 years old (mid-age)^28^, in the light of the social age concept; and parity was classified as the number of births for a given woman, counting a multiple birth pregnancy as one; stillbirths were not considered^29^. Participants’ home addresses were georeferenced using ArcGIS Online World Geocoding Service and Google Earth, due to their superior positional accuracy^30^. Coordinates were used to classify the area of residence as urban, sub-urban, and rural, using the Portuguese National Institute of Statistics^31^ and to identify the neighborhood deprivation. Sub-urban and rural areas were combined in a single category designated of sub-urban/rural.

Finally, alcohol consumption was classified as “Yes” if women reported alcoholic beverages intake, during pregnancy or in the 3 months preceding pregnancy, even if occasionally.

#### Socioeconomic variables

Socioeconomic status, defined as maternal educational level, working status and neighborhood deprivation was estimated. Educational level was categorized as 9 years or less, 10–12, and 13 years of education or higher according to the Portuguese education system. In line with an earlier study^16^, maternal working status at the time of the survey was classified as student, housewife, unemployed and employed. For employed women, occupations were then classified as manual or non-manual^32^. Housewives and unemployed were combined in a single category due to the small number of housewife participants’ after confirmation that these groups were homogeneous for study variables (smoking, smoking cessation, and ETS exposure). Regarding neighborhood deprivation, herein, an updated version of the European Deprivation Index for Portuguese small-areas (EDI-PT) was used^33^. Briefly, this indicator is based on data from the 2011 European Union–Statistics on Income and Living Conditions Survey (EU-SILC) making use of variables that were common to both the EU-SILC and the 2011 Portuguese Population and Housing census. The EDI-PT was established for the smallest area unit (neighborhood, with an average population of 584 inhabitants) and resulted from the weighted sum of the eight selected variables. Neighborhood deprivation was categorized using tertiles: tertile 1 (least deprived); tertile 2 (medium deprived) and tertile 3 (most deprived).

#### Smoking and environmental tobacco smoke exposure

In this study, women were assumed as current smokers if they reported smoking at least one cigarette (or any other smoking products) per day; and considered never smokers if they reported never smoking in their lifetime. Former smokers were defined as those who reported smoking in their lifetime, but who had quit smoking^34^. Further information on the age of smoking initiation and timing of cessation (for former smokers) were also obtained. Smoking status was defined for two different time points: before pregnancy and at birth. At pregnancy, mothers were defined as former smoker if cessation occurred at least 3 months before their pregnancy. Continuation of smoking into pregnancy was analyzed on the basis of cessation in each considered trimester^35^; for this, three additional categories were appraised: (1) quit in the 1^st^ trimester; (2) quit in the 2^nd^ trimester and (3) quit in the 3^rd^ trimester. All those quitting during pregnancy were considered former smokers at birth.

Based on existing knowledge there is no standard procedure for assessment ETS exposure. Thus, in the current study, never-smokers and former smokers were asked to report the duration and frequency of exposure to ETS at home, work and leisure places in the 3 months prior to the pregnancy, and in the 1^st^, 2^nd^ and 3^rd^ trimesters. Response options included: “Never”; “Sporadically”; “Daily, less than 1 h”; “Daily, 1–3 h”; “Daily, 3 or more hours”. For statistical analyses, responses were dichotomized as “Yes” and “No” and participants were further classified as exposed to ETS and not exposed to ETS, respectively.

### Statistical analysis

Results are expressed as means and standard deviations, or as counts and percentages. The distribution of smoking, smoking cessation and ETS exposure according to maternal SEP indicators was examined using the chi-square test or likelihood ratio test, whenever more than 20% of the frequencies found in the crosstab were below 5. Distribution of age of smoking initiation was analyzed using non-parametric Kruskal–Wallis test since data did not assume a normal distribution. A *p*-value < 0.05 was considered statistically significant.

Binary logistic regression analysis was used to explore the prediction of smoking at birth, smoking cessation and ETS exposure in the 3^rd^ trimester among pregnant women using both demographic and SEP variables. First, univariate logistic regression models were used for each outcome variable. Variables that proved to be significant at the univariate analysis (*p* < 0.05) were considered for a multivariate logistic regression analysis, in which significant indicators were entered simultaneously. No significant interactions were found between smoking and smoking cessation, and ETS exposure and SEP variables. Associations were presented as crude and adjusted odds ratio (OR) with 95% confidence intervals (95% CI). All analyses were conducted using the Statistical Package for Social Sciences (SPSS, version 26).

### Ethical approval and data protection

The project was approved by the Ethical Committee of CHSJ, Porto, Portugal (reference nº. 326/16) and was conducted in compliance with the Declaration of Helsinki and its amendments. Before enrolment, each participant received detailed information about the project objectives and risks and benefits of their participation. All those that agreed to participate provided written informed consent. All data were handled and stored anonymously.

## Results

### Study population characteristics

A total of 619 pregnant women were included in this study. Table 1 summarizes the main characteristics of the studied population. At the time of interview, the majority of pregnant women were older than 25 years old (mean age = 31.4 years; range: 18–46), and 45.2% (n = 280) of them already had children. More participants lived in urban areas than in sub-urban/rural areas (95.0% *vs*. 5.0%). Only 78 women (12.6%) of the 619 respondents reported drinking at least some alcohol during pregnancy or in the 3 preceding months.

**Table 1:** Characteristics of pregnant women included in the study (n = 619).

	N	%
**Age [years (mean ± SD, min.-max.)]**	619	31.4 ± 5.6 (18–46)
≤ 25	106	17.1
> 25	513	82.9
Parity*		
0	338	54.6
≥ 1	280	45.2
Area of residence**		
Sub-urban/rural area	31	5.0
Urban area	586	95.0
**Alcohol consumption**		
No	541	87.4
Yes	78	12.6
**Educational level**		
0–9 years	159	25.7
10–12 years	219	35.4
≥ 13 years	241	38.9
**Working status**		
Unemployed/housewives	128	20.7
Students	12	1.9
Employed	479	77.4
Manual	211	44.1
Non-manual	268	55.9
Neighborhood deprivation***		
Least deprived	198	32.0
Medium deprived	199	32.1
Most deprived	198	32.0

*1 missing value; **2 missing values; ***24 missing values.

The proportions of mothers with 9 years or less, 10–12 years and at least 13 years of school education were 25.7%, 35.4% and 38.9%, respectively. Among all participants, most of them were employed (77.4% of the total; of these, 44.1% in manual and 55.9% in non-manual jobs). Neighborhood deprivation was assessed according to their level of socioeconomic deprivation (least-medium-most deprived); approximately one-third of the participants resided in the each of these levels of deprivation.

### Prevalence of smoking, smoking cessation and ETS exposure

Table 2 indicates that before pregnancy, out of the 619 women who participated in this study, 366 (59.1%) women had never been smokers, 13.3% (n = 82) reported to be former smokers, and 171 (27.6%) women were smokers. At birth, the number of current smokers had decreased to 90 (14.6%) participants. Notably, cigarettes were the only tobacco product consumed by smoking women.

**Table 2:** Self-reported maternal smoking, smoking cessation and exposure to environmental tobacco smoke (ETS) according to educational level, working status and neighborhood deprivation.

	Total	Educational level			*p* value	Working status				*p* value	**Neighborhood deprivation**			***p value***
		0–9 years	10–12 years	≥ 13 years		Housewives/unemployed	Students	Employed			Least deprived	Medium deprived	Most deprived	
								Manual	Non-manual					
**Smoking**														
Smoking initiation*														
Age (years)	16.5 ± 3.5	15.8 ± 3.4	16.3 ± 3.3	17.8 ± 3.7	** < 0.001**	15.5 ± 3.6	15.0 ± 1.8	16.6 ± 3.3	17.3 ± 3.6	** < 0.001**	16.8 ± 3.7	16.4 ± 3.5	16.4 ± 3.5	0.768
*Smoking status before pregnancy*														
Never smokers	366 (59.1%)	72 (19.7%)	121 (33.0%)	173 (47.3%)	** < 0.001**	60 (16.4%)	8 (2.2%)	119 (32.5%)	179 (48.9%)	** < 0.001**	122 (35.2%)	118 (34.0%)	107 (30.8%)	0.550
Former smokers	82 (13.3%)	19 (23.2%)	28 (34.1%)	35 (42.7%)		14 (17.1%)	1 (1.2%)	26 (31.7%)	41 (50.0%)		21 (27.2%)	26 (33.8%)	30 (39.0%)	
Current smokers	171 (27.6%)	68 (39.8%)	70 (40.9%)	33 (19.3%)		54 (31.6%)	3 (1.7%)	66 (38.6%)	48 (28.1%)		55 (32.2%)	55 (32.2%)	61 (35.6%)	
*Smoking status at birth*														
Never smokers	366 (59.1%)	72 (19.7%)	121 (33.1%)	173 (47.2%)	** < 0.001**	60 (16.4%)	8 (2.2%)	119 (32.5%)	179 (48.9%)	** < 0.001**	122 (35.2%)	118 (34.0%)	107 (30.8%)	0.638
Former smokers	163 (26.3%)	42 (25.8%)	64 (39.3%)	57 (34.9%)		34 (20.9%)	2 (1.2%)	56 (34.4%)	71 (43.5%)		48 (30.4%)	51 (32.3%)	59 (37.3%)	
Current smokers	90 (14.6%)	45 (50.0%)	34 (37.8%)	11 (12.2%)		34 (37.8%)	2 (2.2%)	36 (40.0%)	18 (20.0%)		28 (31.1%)	30 (33.3%)	32 (35.6%)	
Cessation rate**														
No cessation	90 (52.3%)	45 (50.0%)	34 (37.8%)	11 (12.2%)	**0.012**^**#**^	34 (37.8%)	2 (2.2%)	36 (40.0%)	18 (20.0%)	0.257^#^	28 (31.1%)	30 (33.3%)	32 (35.6%)	0.144^#^
Before pregnancy	7 (4.1%)	2 (28.6%)	2 (28.6%)	3 (42.8%)		1 (14.3%)	0	2 (28.6%)	4 (57.1%)		1 (14.2%)	3 (42.9%)	3 (42.9%)	
1^st^ trimester	59 (34.3%)	15 (25.4%)	26 (44.1%)	18 (30.5%)		13 (22.0%)	1 (1.7%)	22 (37.3%)	23 (39.0%)		23 (39.0%)	15 (25.4%)	21 (35.6%)	
2^nd^ trimester	8 (4.6%)	5 (62.5%)	3 (37.5%)	0		4 (50.0%)	0	3 (37.5%)	1 (12.5%)		0	4 (50.0%)	4 (50.0%)	
3^rd^ trimester	3 (1.7%)	1 (33.3%)	2 (66.7%)	0		1 (33.3%)	0	2 (66.7%)	0		1 (33.3%)	2 (66.7%)	0	
Exposure to ETS***														
*Before pregnancy*														
No	191 (42.6%)	32 (16.8%)	53 (27.7%)	106 (55.5%)	**0.004**	27 (14.1%)	4 (2.1%)	54 (28.3%)	106 (55.5%)	0.130	62 (34.1%)	66 (36.2%)	54 (29.7%)	0.550
Yes	257 (57.4%)	59 (23.0%)	96 (37.3%)	102 (39.7%)		47 (18.3%)	5 (1.9%)	91 (35.4%)	114 (44.4%)		81 (33.5%)	78 (32.2%)	83 (34.3%)	
*1st trimester*														
No	222 (48.8%)	37 (16.7%)	60 (27.0%)	125 (56.3%)	** < 0.001**	30 (13.5%)	4 (1.8%)	64 (28.8%)	124 (55.9%)	**0.049**	68 (32.4%)	80 (38.1%)	62 (29.5%)	0.208
Yes	233 (51.2%)	56 (24.0%)	91 (39.1%)	86 (36.9%)		45 (19.3%)	5 (2.2%)	83 (35.6%)	100 (42.9%)		76 (34.4%)	67 (30.3%)	78 (35.3%)	
*2nd trimester*														
No	250 (48.6%)	41 (16.4%)	74 (29.6%)	135 (54.0%)	** < 0.001**	36 (14.4%)	5 (2.0%)	73 (29.2%)	136 (54.4%)	**0.042**	81 (34.2%)	87 (36.7%)	69 (29.1%)	0.149
Yes	264 (51.4%)	67 (25.4%)	103 (39.0%)	95 (35.6%)		52 (19.7%)	5 (1.9%)	96 (36.4%)	111 (52.0%)		86 (34.0%)	75 (29.6%)	92 (36.4%)	
*3rd trimester*														
No	262 (50.2%)	44 (16.8%)	77 (29.4%)	141 (53.8%)	** < 0.001**	41 (15.7%)	5 (1,9%)	74 (28.2%)	142 (54.2%)	**0.022**	86 (34.7%)	92 (37.1%)	70 (28.2%)	0.053
Yes	260 (49.8%)	69 (26.5%)	103 (39.6%)	88 (33.9%)		51 (19.6%)	5 (1.9%)	98 (37.7%)	106 (40.8%)		81 (32.4%)	74 (29.6%)	95 (38.0%)	

Row percentages are presented in all cases, except for total, that indicates column percentages.

*Mean ± SD; **4 missing values; ***ETS exposure was calculated considering non-smokers and former smokers at each time point.

^**#**^Likelihood ratio test instead of chi-square test.

Data also shows that cessation occurred before pregnancy for 4.1% of smokers (n=7), and during pregnancy for 40.6% of them (n = 70). To note that, 90 (52.3%) women have never stopped tobacco use.

Concurrently, the prevalence of ETS exposure among non-smoking pregnant women (including former smokers) was found to decrease from 57.4% before pregnancy to 51.2% in the 1^st^ trimester, 51.4% in the 2^nd^ trimester, and 49.8% in the 3^rd^ trimester; this represents an increment in the number of women who reported no ETS exposure at home, leisure places or at work, along the three trimesters of pregnancy.

Self-reported smoking, smoking cessation and ETS exposure by educational level, working status and neighborhood deprivation is presented in Table 2. The average age of smoking initiation was found to be higher as maternal educational level increased (*p* < 0.001). Likewise, early smoking initiation varied significantly according to working status (*p* < 0.001); the students and housewives/unemployed being the earlier initiators. No differences were observed for neighborhood deprivation (*p* = 0.768).

The distribution of never smokers, former smokers and current smokers, both before pregnancy and at birth, was also found to be significantly different depending on the educational level (*p* < 0.001) and working status (*p* < 0.001); the majority of smoking pregnant women were found to have lower educational levels (< 13 years) and were either working on a manual job or were unemployed/housewives. No differences were observed regarding neighborhood deprivation before pregnancy (*p* = 0.550). As expected, since women did not move to a new home, similar results were obtained at birth (*p* = 0.638). Concerning smoking cessation, continuers and quitters significantly differed in educational level (*p* < 0.012), but not in working status (*p* = 0.257) or neighborhood deprivation (*p* = 0.144), being those who had more than 13 years of education more likely to quit smoking compared with mothers who had been educated for less than 12 years.

Self-reported ETS exposure among never and former smokers differed significantly according to educational level at all-time points considered (*p* = 0.004 before pregnancy and *p* < 0.001 throughout pregnancy) and working status during pregnancy (*p* = 0.049 in the 1^st^, *p* = 0.042 in the 2^nd^ and *p* = 0.022 in the 3^rd^ trimester); women with higher educational level and working in non-manual jobs were found to be less exposed. Detailed information on the duration and frequency of ETS exposure throughout pregnancy is presented in Table 1S.

### Associations between demographic and SEP variables and smoking

Table 3 presents the results of unadjusted and adjusted models predicting maternal smoking from demographic and SEP variables. The results obtained with the model adjusted for variables found to be significant at the univariate model show a significant association between age and smoking (OR 0.554; 95%CI 0.314–0.978) as well as between educational level and smoking (OR 0.488; 95%CI 0.286–0.831 for 10–12 years, and OR 0.156; 95%CI 0.066–0.371 for more than 13 years).

**Table 3:** Crude and adjusted logistic regression analysis of demographic and socioeconomic status variables on smoking and environmental tobacco smoke (ETS) exposure.

	Smoking		ETS exposure	
	OR (95%CI)	aOR (95%CI)^§^	OR (95%CI)	aOR (95%CI)^¥^
**Maternal age**				
≤ 25	Reference	Reference	Reference	–
> 25	0.336 (0.203–0.554)**	0.554 (0.314–0.978)*	0.621 (0.379–1.020)	–
**Parity**				
0	Reference	–	Reference	–
≥ 1	1.123 (0.718–1.758)	–	0.811 (0.575–1.143)	–
**Area of residence**				
Sub-urban/rural area	Reference	–	Reference	–
Urban area	1.627 (0.484–5.469)	–	1.382 (0.641–2.982)	–
**Alcohol consumption**				
No	Reference	–	Reference	–
Yes	1.079 (0.558–2.089)	–	1.489 (0.882–2.513)	–
**Educational level**				
0–9 years	Reference	Reference	Reference	Reference
10–12 years	0.466 (0.282–0.770)*	0.488 (0.286–0.831)*	0.882 (0.547–1.424)	0.887 (0.542–1.450)
≥ 13 years	0.121 (0.060–0.243)**	0.156 (0.066–0.371)**	0.397 (0.250–0.629)**	0.397 (0.218–0.729)*
**Working status**				
Unemployed/housewives	Reference	Reference	Reference	Reference
Students	0.553 (0.115–2.653)	0.413 (0.080–2.133)	0.774 (0.210–2.852)	0.802 (0.212–3.032)
Manual	0.569 (0.334–0.968)*	0.576 (0.327–1.017)	1.056 (0.637–1.751)	0.897 (0.533–1.511)
Non-manual	0.199 (0.107–0.370)**	0.591 (0.276–1.264)	0.588 (0.365–0.949)*	0.920 (0.530–1.597)
**Neighborhood deprivation**				
Least deprived	Reference	–	Reference	–
Medium deprived	1.078 (0.617–1.882)	-	0.857 (0.559–1.313)	–
Most deprived	1.170 (0.675–2.029)	–	1.404 (0.913–2.159)	–

OR, odds ratio; aOR, adjusted odds ratio; CI, confidence interval.

*: *p* < 0.05; **: *p* < 0.001.

^§^Adjusted for age, educational level and working status.

^¥^Adjusted for educational level and working status.

### Associations between demographic and SEP variables and ETS exposure

According to findings presented in Table 3, in the adjusted model, those with less than 13 years of education were more likely to be exposed to ETS. Thus, university or equivalent level as the highest educational level (OR 0.397; 95%CI 0.218–0.729) was associated with decreased odds of exposure to ETS.

### Associations between demographic and SEP variables and smoking cessation

Table 4 summarizes the results of unadjusted and adjusted analyses to characterize smoking cessation by demographic and SEP variables. In line with the observed for smoking and ETS exposure, adjusted logistic regression model showed that pregnant women with higher educational level were more likely to quit consumption (OR 2.969; 95% CI 1.037–8.506).

**Table 4:** Crude and adjusted logistic regression analysis of demographic and socioeconomic status variables on smoking cessation.

	OR (95%CI)	aOR (95%CI)^§^
**Maternal age**		
≤ 25	Reference	–
> 25	1.795 (0.896–3.596)	–
**Parity**		
0	Reference	–
≥ 1	0.639 (0.343–1.193)	–
**Area of residence**		
Sub-urban/rural area	Reference	–
Urban area	2.651 (0.270–26.026)	–
**Alcohol consumption**		
No	Reference	–
Yes	1.818 (0.807–4.097)	–
**Educational level**		
0–9 years	Reference	Reference
10–12 years	1.857 (0.926–3.722)	1.678 (0.816–3.451)
≥ 13 years	3.652 (1.505–8.865)**	2.969 (1.037–8.506)*
**Working status**		
Unemployed/housewives	Reference	Reference
Students	0.895 (0.076–10.528)	1.022 (0.085–12.294)
Manual	1.483 (0.703–3.128)	1.536 (0.716–3.295)
Non-manual	2.784 (1.231–6.295)*	1.786 (0.712–4.485)
**Neighborhood deprivation**		
Least deprived	Reference	–
Medium deprived	0.864 (0.402–1.855)	–
Most deprived	0.945 (0.449–1.988)	–

OR, odds ratio; aOR, adjusted odds ratio; CI, confidence interval.

*p < 0.05; **p < 0.001.

^§^Adjusted for educational level.

## Discussion

Smoking and ETS exposure are associated with several immediate and long-term health effects on both pregnant women and their newborns^5,36^. Understanding the trends and determinants of these behaviors is crucial for the development of effective preventive interventions that aim to reduce the associated burden of disease. In this context, the role of health professionals assumes particular importance and can constitute an efficacious preventive health action as they can offer the best behavioral counseling and/or support for smoking cessation during, and preferably also after, pregnancy; as well as recommend the creation of a smoke free home, including post-discharge from delivery.

In Portugal, there are no previous studies on ETS exposure during pregnancy, and information available on smoking and smoking cessation among pregnant women has not been reported in detail since 2013, in a study that examines data from 2005 to 2006^16^. Meanwhile, Portugal has surpassed different societal and economical challenges, and new legislation to promote tobacco cessation and smoke-free environments has been implemented (namely, Lei 37/2007^10^ and tax and prices measures, as defined in Decreto-Lei 73/2010^37^). The latest population estimates from 2014 shows that Portuguese women were at that time in stage 2 of the tobacco epidemic model, and, therefore, its peak had not yet been reached^4^.

This study found that the prevalence of smoking before pregnancy (27.7%) was higher than in the previous Portuguese study from 2013, where the prevalence was 22.9%^16^. Even though the most recent estimate on smoking prevalence among Portuguese women has been set in 14.6%, one must keep in mind that our study includes only women of fertile age, and that these are the groups presenting higher prevalence of smokers^4^. Furthermore, women included in this study were mainly from urban areas, a factor that may be associated with increased smoking frequency^4^; some authors have highlighted that social and cultural dimensions of lifestyle could account for rural–urban differences^38^.

At birth, results showed that the prevalence of smoking women was 14.6%, which is very close to what has been recently reported in Portugal (14%)^17^, and slightly higher than the described by Alves et al.^16^ (12%). The prevalence at delivery found in this study is relatively high when compared to other countries, such as Denmark (6%)^11^, USA (7.1%)^22^, and China (3.8%)^25^. This discrepancy could be attributed to differences in study design, sample selection, variable definition, but also to tobacco control policies of each country, and population awareness for tobacco effects^39^. Herein, more than half of the smoking women (52.3%) did not stop smoking neither in the 3 months before pregnancy, nor during gestation. In accordance with our findings, Alves et al.^16^, in Portugal, reported a similar cessation rate (52.6%), showing that there was no significant progression on this matter in the last 15 years.

In line with prior research on the role of demographic characteristics on maternal consumption in pregnancy^11,23,25,40^, the current study suggests that mid-age women (older than 25 years-old), probably due to individual life circumstances and higher understanding of harms related to smoking, are less likely to smoke. Other relevant predictors of smoking among pregnant women identified in previous studies include parity^11,24^, and alcohol consumption^13,23^. Nevertheless, in our study, after adjustment for significant variables, there was no significant association between parity or alcohol use and smoking status.

As reported by other authors^11,13,22^, in our study, educational disadvantage was found to be a predictor of smoking status during pregnancy; participants who belong to the highest educational levels had a lower probability of smoking. In Portugal, these inequalities in pregnant smoking women have also been previously described^16^. Educational level may not only increase the likelihood of awareness of harmful effects of smoking during pregnancy^41^, but also of access to cessation programs^42^. In fact, several studies have noted that more highly educated women were more likely to quit smoking during pregnancy^11,22,43^. In our findings, a significant association was also found between educational levels (an indicator of SEP) and smoking cessation, indicating that continuers were more often those with lower education and with unskilled work.

In the current study, findings concerning working status, even though not significant, align with those in the literature, that report occupation as a predictor for smoking before and during pregnancy^11,13,26^; occupation is also commonly related to an individual education and income^44^. Research also supports that smoking is a habituated response to the circumstance of housewife, often associated with economic vulnerability^45^.

Altogether, these findings evidence that women maintain their smoking habits even after learning that they are pregnant; however, during pregnancy, women are particularly responsive to accessible tobacco cessation interventions^46,47^, and therefore it is crucial to devote increased efforts on effective targeted smoking prevention and cessation strategies in this period of life, in the frame of the already existing national tobacco control plan and smoking cessation guidelines especially in Portugal.

Another important finding of the current study relates to the characterization of ETS exposure during pregnancy, carried out in Portugal for the first time. Results obtained showed that more than half of non-smokers participants reported to be exposed to ETS before pregnancy (57.4%) and that this number decreases with pregnancy progression. This prevalence estimate is lower than that described by other authors^48^, but higher when compared to a recent study carried out by Alghamdi et al.^49^. In fact, the prevalence here obtained seems high if one considers that in 2015, it was estimated that only 12.8% of the total Portuguese population reported to be exposed to ETS^8^. In Portugal, a smoke-free law was implemented in 2008 (Lei 37/2007^10^) in order to decrease exposure to ETS and its effects among non-smokers. Nevertheless, residential homes and private vehicles are not covered in the mentioned law, what may justify the high value here observed; these results suggest that ETS exposure may constitute a serious negative health outcome for pregnant women and developing fetus and, thus, it is essential to strengthen people's awareness of active and passive tobacco hazards.

Our results also indicate that exposure to ETS tended to be associated with participants who were less educated, as has been demonstrated by other authors^8,49^; participants with high levels of literacy are more likely to engage in health-promoting behaviors^42^. These data suggest that it is essential to strength people’s awareness regarding tobacco hazards and to implement more comprehensive smoke-free laws and legislations to protect particularly the less educated individuals from ETS.

Possible limitations in this study include selection and recall bias, lack of data on income and smoking status of the partner prior and during pregnancy. Another inherent limitation of this study comes from the representativeness of the study population that was not nationally representative. The sample consisted of pregnant women from the Porto Metropolitan Area, in the north of Portugal, which could differ from the general birthing Portuguese population or even from those pregnant women living in other Portuguese geographic regions (urban or non-urban areas). Therefore, the generalizability of the obtained findings should be considered when interpreting the results. By recruiting volunteers to a study on smoking and its health effects, it is more likely to have smokers aware of smoking hazards as participants. In addition, subject categorization as smoker or non-smoker was based on self-reported information; ideally, this should have been confirmed with biomarkers, such as urinary cotinine levels. Nonetheless, qualitative measures are routinely used in many national and international health behavioral surveillance studies, providing accurate results as previously described by e.g. Bernstein et al.^50^ and Mattsson et al.^51^. Recall bias, on the other hand, was kept minimal due to the short-time relapse from pregnancy to data collection. Information on income has not been collected but many studies have shown that this indicator is closely related to education and occupation^52^. In opposition, we have no proxy variable for smoking status of partners, which is known to have a significant influence on continuation^23,24^ and smoking cessation behaviors^23^. In addition, information whether the participants were asked by their health care provider, about advice and/or support on non-pharmacological strategies or pharmacological interventions, or both, to quit smoking should be collected in future epidemiological studies. On the other hand, this is a comprehensive study that not only includes a large number of participants but also contains variables that have not yet been examined before in Portugal, such as data for each trimester of pregnancy, alcohol use and neighborhood deprivation. In addition, by collecting information on ETS exposure in different indoor settings prior or during pregnancy made it possible to explore, for the first time in Portugal, the exposure to ETS among pregnant women.

In conclusion, our findings show that the smoking prevalence and ETS exposure remain a current challenge in Portugal, with 27.6% of smoking women before pregnancy, 14.6% of smoking women at delivery and 49.8% of non-smoking pregnant women exposed to ETS in the 3^rd^ trimester. There is also a low rate of attempting to quit smoking among pregnant women (52.3%). Furthermore, individual SEP, assessed by educational level, was found to be predictive of smoking, smoking cessation and ETS exposure, which shows that further efforts on preventive and interventional measures to reduce smoking among the less educated are still of need. Therefore, public policies must continue to consider the reduction in inequalities in smoking among the most vulnerable social groups to, also, prevent future health inequalities.

## Supplementary information

Supplementary information.

## Data Availability

The datasets analyzed during the current study are available from the corresponding author on reasonable request.
