# Supplementary material for: The importance of socioeconomic position in smoking, cessation and environmental tobacco smoke exposure during pregnancy
Source: Sci Rep. 2020 Sep 24;10:15584. doi: 10.1038/s41598-020-72298-8 (PMC7515871; doi:10.1038/s41598-020-72298-8)
Supplement: Supplementary file 1 — Supplementary information. [file 41598_2020_72298_MOESM1_ESM.docx]

**The importance of socioeconomic position in smoking, cessation and environmental tobacco smoke exposure during pregnancy**

Joana Madureira^1,2,#^, Alexandra Camelo^1,2,#^, Ana Inês Silva^1,2,3^, Ana Teresa Reis^1,2^, Filipa Esteves^1,2^, Ana Isabel Ribeiro^2^, João Paulo Teixeira^1,2, *^, Carla Costa^1,2^

^#^ Both authors contributed equally to this manuscript

^1^Environmental Health Department, National Institute of Health, Rua Alexandre Herculano 321, 4000-055 Porto, Portugal; ^2^EPIUnit-Instituto de Saúde Pública, Universidade do Porto, Rua das Taipas 135, 4050-600 Porto, Portugal; ^3^ICBAS-Institute of Biomedical Sciences Abel Salazar, U. Porto-University of Porto, Rua de Jorge Viterbo Ferreira 228, 4050-313 Porto, Portugal

**^*^**corresponding author: [jpft12@gmail.com](mailto:jpft12@gmail.com)

| **Table 1S.** Detailed duration and frequency of self-reported exposure to environmental tobacco smoke throughout pregnancy, according to educational level, working status and neighborhood deprivation. | | | | | | | | | | | | | | |
| --- | --- | --- | --- | --- | --- | --- | --- | --- | --- | --- | --- | --- | --- | --- |
|  | **Total** | **Educational level** | | | ***p* value** | **Working Status** | | | | ***p* value** | **Neighborhood deprivation** | | | ***p* value** |
|  |  | 0-9 years | 10-12 years | ≥13 years |  | Housewives/ Unemployed | Students | Employed | |  | Least deprived | Medium deprived | Most deprived |  |
|  |  |  |  |  |  |  |  | Manual | Non-manual |  |  |  |  |  |
| **Before Pregnancy** | | | | | | | | | | | | | | |
| No | 191 (42.6%) | 32 (16.8%) | 53 (27.7%) | 106 (55.5%) | **<0.001** | 27 (14.1%) | 4 (2.1%) | 54 (28.3%) | 106 (55.5%) | **0.032^#^** | 62 (34.1%) | 66 (36.2%) | 54 (29.7%) | 0.878 |
| Sporadic | 118 (26.3%) | 20 (17,0%) | 38 (32.2%) | 60 (50.8%) |  | 13 (11.0%) | 2 (1.7%) | 41 (34.8%) | 62 (52.5%) |  | 36 (34.3%) | 35 (33.3%) | 34 (32.4%) |  |
| Daily, less than 3 hours | 110 (24.6%) | 31 (28.2%) | 49 (44.5%) | 30 (27.3%) |  | 30 (27.3%) | 2 (1.8%) | 38 (34.5%) | 40 (36.4%) |  | 34 (31.5%) | 36 (33.3%) | 38 (35.2%) |  |
| Daily, more than 3 hours | 29 (6.5%) | 8 (27.6%) | 9 (31.0%) | 12 (41.4%) |  | 4 (13.8%) | 1 (3.4%) | 12 (41.4%) | 12 (41.4%) |  | 11 (37.9%) | 7 (24.2%) | 11 (37.9%) |  |
| **1^st^ Trimester** | | | | | | | | | | | | | | |
| No | 222 (48.8%) | 37 (16.7%) | 60 (27.0%) | 125 (56.3%) | **<0.001** | 30 (13.5%) | 4 (1.8%) | 64 (28.8%) | 124 (55.9%) | **0.005^#^** | 68 (32.4%) | 80 (38.1%) | 62 (29.5%) | 0.515 |
| Sporadic | 104 (22.9%) | 17 (16.4%) | 36 (34.6%) | 51 (49.0%) |  | 13 (12.5%) | 2 (1.9%) | 33 (31.7%) | 56 (53.9%) |  | 36 (38.3%) | 28 (29.8%) | 30 (31.9%) |  |
| Daily, less than 3 hours | 106 (23.3%) | 31 (29.3%) | 47 (44.3%) | 28 (26.4%) |  | 30 (28.3%) | 2 (1.9%) | 39 (36.8%) | 35 (33.0%) |  | 31 (29.8%) | 33 (31.7%) | 40 (38.5%) |  |
| Daily, more than 3 hours | 23 (5.0%) | 8 (34.8%) | 8 (34.8%) | 7 (30.4%) |  | 2 (8.7%) | 1 (4.4%) | 11 (47.8%) | 9 (39.1%) |  | 9 (39.1%) | 6 (26.1%) | 8 (34.8%) |  |
| **2^nd^ Trimester** | | | | | | | | | | | | | | |
| No | 250 (48.7%) | 41 (16.4%) | 74 (29.6%) | 135 (54.0%) | **<0.001** | 36 (14.4%) | 5 (2.0%) | 73 (29.2%) | 136 (54.4%) | **0.012^#^** | 81 (34.2%) | 87 (36.7%) | 69 (29.1%) | 0.400 |
| Sporadic | 120 (23.3%) | 22 (18.3%) | 42 (35.0%) | 56 (46.7%) |  | 16 (13.3%) | 2 (1.7%) | 43 (35.8%) | 59 (49.2%) |  | 41 (37.3%) | 32 (29.1%) | 37 (33.6%) |  |
| Daily, less than 3 hours | 120 (23.3%) | 36 (30.0%) | 51 (42.5%) | 33 (27.5%) |  | 34 (28.3%) | 2 (1.7%) | 42 (35.0%) | 42 (35.0%) |  | 35 (29.4%) | 37 (31.1%) | 47 (39.5%) |  |
| Daily, more than 3 hours | 24 (4.7%) | 9 (37.5%) | 10 (41.7%) | 5 (20.8%) |  | 2 (8.3%) | 1 (4.2%) | 11 (45.8%) | 10 (41.7%) |  | 10 (41.7%) | 6 (25.0%) | 8 (33.3%) |  |
| **3^rd^ Trimester** | | | | | | | | | | | | | | |
| No | 262 (50.2%) | 44 (16.8%) | 77 (29.4%) | 141 (53.8%) | **<0.001** | 41 (15.7%) | 5 (1.9%) | 74 (28.2%) | 142 (54.2%) | **0.003^#^** | 86 (34.7%) | 92 (37.1%) | 70 (28.2%) | 0.168 |
| Sporadic | 117 (22.4%) | 23 (19.7%) | 41 (35.0%) | 53 (45.3%) |  | 14 (12.0%) | 2 (1.7%) | 44 (37.6%) | 57 (48.7%) |  | 38 (35.2%) | 33 (30.5%) | 37 (34.3%) |  |
| Daily, less than 3 hours | 122 (23.4%) | 37 (30.3%) | 53 (43.5%) | 32 (26.2%) |  | 35 (28.7%) | 2 (1.6%) | 43 (35.3%) | 42 (34.4%) |  | 34 (28.1%) | 37 (30.6%) | 50 (41.3%) |  |
| Daily, more than 3 hours | 21 (4.0%) | 9 (42.9%) | 9 (42.9%) | 3 (14.2%) |  | 2 (9.5%) | 1 (4.8%) | 11 (52.4%) | 7 (33.3%) |  | 9 (42.9%) | 4 (19.0%) | 8 (38.1%) |  |

Note: Row percentages are presented in all cases, except for total, that indicates column percentages; **^#^**likelihood ratio test instead of chi-square test.
